# Known Cellular and Receptor Interactions of Animal and Human Coronaviruses: A Review

**DOI:** 10.3390/v14020351

**Published:** 2022-02-08

**Authors:** Holly Everest, Phoebe Stevenson-Leggett, Dalan Bailey, Erica Bickerton, Sarah Keep

**Affiliations:** 1 The Pirbright Institute, Surrey GU24 0NF, UK; holly.everest@pirbright.ac.uk (H.E.); phoebe.stevenson-leggett@pirbright.ac.uk (P.S.-L.); dalan.bailey@pirbright.ac.uk (D.B.); erica.bickerton@pirbright.ac.uk (E.B.); 2 Nuffield Department of Medicine, University of Oxford, Oxford OX3 7BN, UK

**Keywords:** coronavirus, receptor-binding, glycan, SARS-CoV-2, sialic acid, omicron, spike protein, haemagglutinin-esterase, cleavage, host interaction

## Abstract

This article aims to review all currently known interactions between animal and human coronaviruses and their cellular receptors. Over the past 20 years, three novel coronaviruses have emerged that have caused severe disease in humans, including SARS-CoV-2 (severe acute respiratory syndrome virus 2); therefore, a deeper understanding of coronavirus host–cell interactions is essential. Receptor-binding is the first stage in coronavirus entry prior to replication and can be altered by minor changes within the spike protein—the coronavirus surface glycoprotein responsible for the recognition of cell-surface receptors. The recognition of receptors by coronaviruses is also a major determinant in infection, tropism, and pathogenesis and acts as a key target for host-immune surveillance and other potential intervention strategies. We aim to highlight the need for a continued in-depth understanding of this subject area following on from the SARS-CoV-2 pandemic, with the possibility for more zoonotic transmission events. We also acknowledge the need for more targeted research towards glycan–coronavirus interactions as zoonotic spillover events from animals to humans, following an alteration in glycan-binding capability, have been well-documented for other viruses such as Influenza A.

## 1. Background

**Figure 1:**
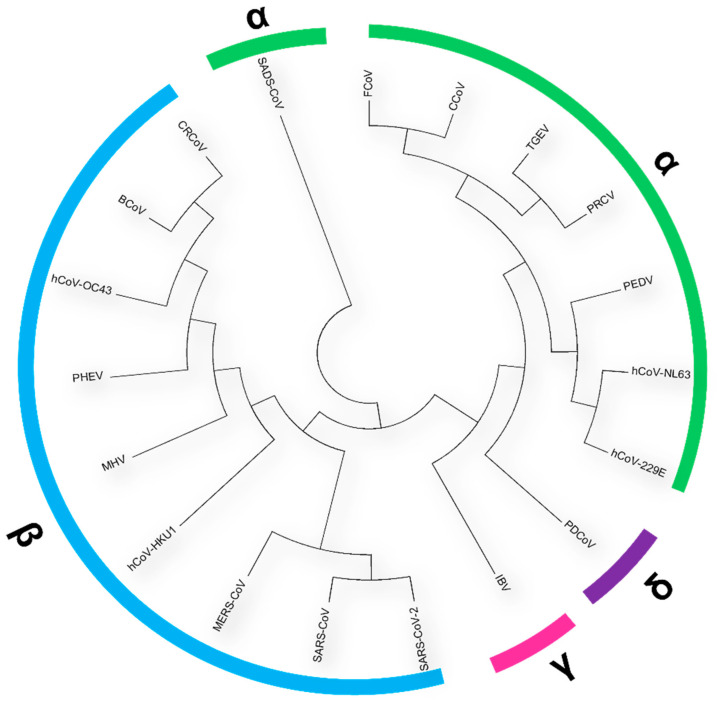
A midpoint rooted circular cladogram of representative coronavirus spike glycoprotein sequences across the *alpha*-, *beta*-, *gamma*- and *delta*-genera. Representative strains from Genbank were used (Accession Numbers outline in Table 1). MUSCLE [22] was used to align the sequences in MEGA X [23]. A Neighbour-Joining cladogram was generated, which highlights the specific coronavirus genera and is coloured accordingly. The genera are also annotated in bold font denoted by Greek symbol (*alpha*-α, *beta*-β, *delta*-δ, *gamma*-γ).

Coronaviruses are a large family of enveloped, positive sense, single stranded RNA viruses [1], which as classified by the International Committee on Taxonomy of Viruses (ICTV) are part of the *Nidovirales* order, sub-order *Coronavirinae*, family *Coronaviridae*. The family is further subdivided into the *orthocoronavirinae* which consists of four genres, *alphacoronavirus*, *betacoronavirus*, *gammacoronavirus* and *deltacoronavirus* [2,3] (Figure 1). The first coronavirus, identified in 1937, was the avian *gammacoronavirus* Infectious Bronchitis Virus (IBV); the aetiological agent of Infectious Bronchitis. It is an acute, highly contagious, economically important respiratory disease of domestic fowl [4]. Transmissible Gastroenteritis Virus (TGEV), which infects swine, was identified over a decade later in the 1940s [5]. Up until the emergence of severe acute respiratory syndrome coronavirus (SARS-CoV) in 2002–2003 [6,7], the coronavirus research field predominantly centred on those of veterinary concern. Interest in the coronavirus family grew exponentially in the aftermath the emergence of SARS-CoV, where the resulting epidemic highlighted that coronavirus infection of humans could result in serious and fatal disease. This led to the identification of many new coronavirus family members, including the human coronavirus hCoV-NL63 [8,9], Beluga Whale coronavirus [10] and several bat coronaviruses, which are reported to cause zoonotic spillover events [11,12].

The outbreaks of SARS-CoV and MERS-CoV in 2012 highlighted the ability of coronaviruses to jump the species barrier causing implications for human health [13,14]. Coronaviruses are currently a hot topic due to the emergence in 2019 of the pandemic *betacoronavirus* severe acute respiratory syndrome coronavirus 2 (SARS-CoV-2), which can cause severe and sometimes fatal respiratory disease in humans [15]. The recent zoonotic emergence of the alphacoronavirus Swine Acute Diarrhoea Syndrome Coronavirus (SADS-CoV), causing outbreaks of severe diarrhoea in suckling piglets [16,17], demonstrates that emerging coronaviruses do not just present a public health concern but also a veterinary health and welfare concern. A major determinant of inter-species transmission, and therefore emerging coronaviruses is receptor-binding capability. This has been comprehensively studied with regard to Influenza A viruses (IAVs) but to a much lesser extent with coronaviruses [18,19,20]. The first coronavirus receptor identified in 1991 was the murine hepatitis virus (MHV) receptor CEACAM1 [21]. Since then, multiple other coronavirus receptors have been identified; however, some remain unknown, with the primary receptor for several coronaviruses including IBV and SADS-CoV yet to be identified.

Receptor identification contributes significantly to our understanding of host, tissue, and cell tropism, and helps explain aspects of virus pathogenesis. This is crucial in understanding zoonotic potential, viral and host adaptation and clinical disease. Furthermore, receptor identification can inform the development of antivirals, vaccines, and diagnostic tests, which have a considerable impact towards both human and animal health [24]. Receptor-binding is a complicated process, with a number of stages involved. Whilst viruses utilise primary receptors for entry, additional molecules can be used for attachment to the cell surface membrane or alternative methods of entry at a later stage. This is elegantly demonstrated with human immunodeficiency virus (HIV) which binds the cellular receptor CD4 [25,26], and then subsequently either the chemokine co-receptor CCR5 or CXCR4 (reviewed by Wilen et al. [27]). Host-cell molecules, such as CD4, that bind virus-attachment proteins and are required for entry are regarded as primary receptors [28]. There are then some molecules that bind specifically to viral proteins and are also required for entry (in addition to just the primary receptor) typically ensuring continuation of the entry process after binding; these are denoted as entry co-factors or attachment factors [28,29]; an example being SARS-CoV-2 and its dependence on heparan sulfate for entry [30]. Some viruses can also utilise receptors in the absence of the primary receptor, typically with a much lower efficiency for entry, these are referred to as alternative virus receptors.

Whilst some aspects of coronavirus receptor interactions have previously been reviewed (Guruprasad et al. [31], Reguera et al. [32], Holmes et al. [33] and others); this review aims to give a more comprehensive and updated overview of all known animal and human coronavirus receptor and cellular interactions as a whole. It covers receptor recognition and entry mechanisms, the spike and haemagglutinin-esterase glycoproteins, receptor-binding domains and primary receptors and attachment factors.

## 2. Receptor Recognition and Entry Mechanisms of Coronaviruses

**Figure 2:**
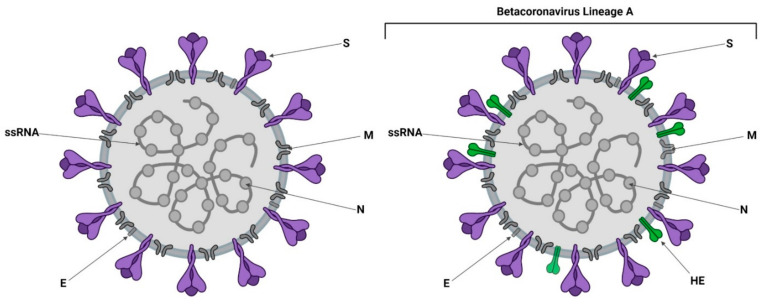
Annotated diagrams of the coronavirus virion—the presence of the HE surface protein differentiates the *betacoronavirus* lineage A viruses (**right**) from other coronaviruses (**left**). The surface proteins are also denoted by colour S glycoprotein (purple) and HE glycoprotein (green). Figure Adapted from “Human Coronavirus Structure”, by BioRender.com (2021). Available online: https://app.biorender.com/biorender-templates (accessed on 2 February 2022).

Coronavirus entry is a multistep process (Figure 2) initiated by the Spike (S) glycoprotein, a large, highly glycosylated type I transmembrane protein, class I fusion protein of ~180 kDa. The S glycoprotein binds to cell surface receptors, attaching the virion to the host cell membrane ([34,35]. Lineage A betacoronaviruses also have a Haemagglutinin-Esterase (HE) glycoprotein, which acts as a receptor destroying enzyme (RDE) [36].

**Figure 3:**
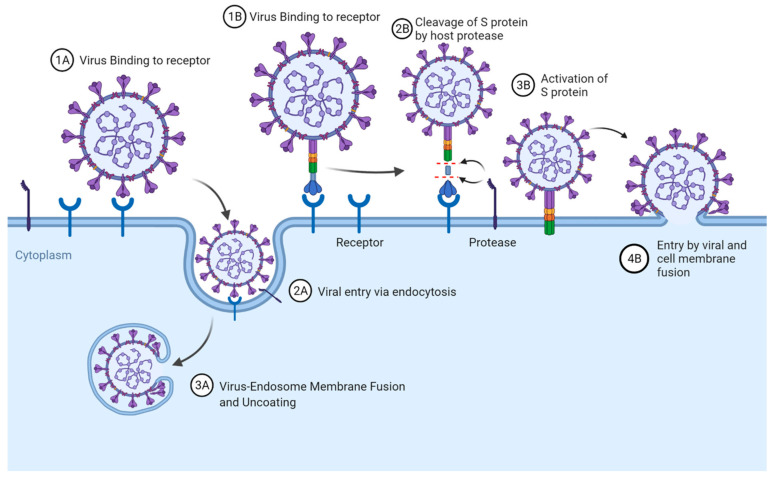
The cellular entry mechanisms of coronaviruses. Route (**A**) = internalisation via endocytosis, Route (**B**) = internalisation via membrane fusion. Figure adapted from the “Coronavirus Replication Cycle” by BioRender.com (2021). Available online: https://app.biorender.com/biorender-templates (accessed on 2 February 2022).

From this point there are then two recognised entry pathways including internalisation via endocytosis with virus to cell fusion taking place in the endosomal compartment [37,38], and internalisation via direct fusion at the plasma membrane [39,40,41,42] (Figure 3).

## 3. The Spike Glycoprotein

**Figure 4:**
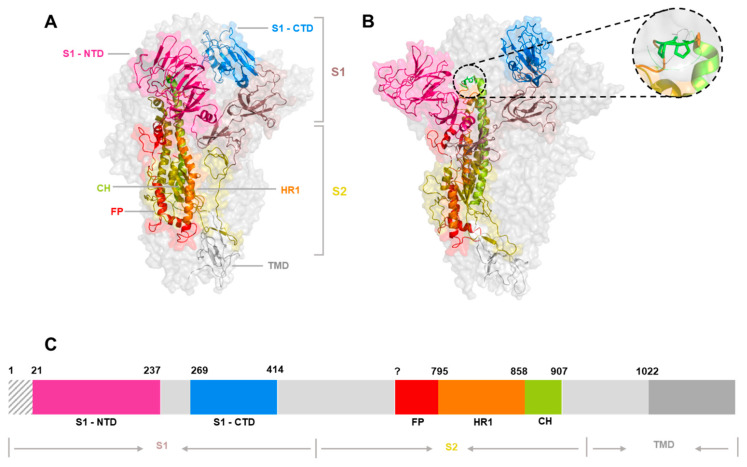
Structure and schematic of IBV S glycoprotein in pre-fusion conformation with 2P stabilisation. (**A**) IBV trimeric structure modelled in PyMol [61] using the Cryo-EM structure of IBV strain M41 [53] (**RCSB PDB**: 6CVO). (**A**) single monomer is highlighted with each region of the S glycoprotein annotated. (**B**) The M41 IBV structure modelled in PyMol with the substitution of two proline amino acid residues allowing for pre-fusion 2P stabilisation. The proline insertions are indicated. (**C**) Linear schematic diagram of IBV spike ectodomain. Question mark indicates that the exact amino acid residue location of the FP is unidentified.

For a comprehensive understanding of coronavirus receptor-binding, it is necessary to understand the role and function of the S glycoprotein, which is categorised into three segments (Figure 4A); an ectodomain, a single-pass transmembrane anchor and a short intracellular tail [43,44]. The ectodomain can be further divided into two subunits—the S1 subunit, which mediates receptor-binding, and the S2 subunit, which mediates virus-to-cell and cell-to-cell fusion [45,46]. Two structurally distinct conformations are recognised; pre- and post-fusion [47,48,49]. The pre-fusion conformation protrudes outwards from the virion and consists of the S1 globular head sat upon the S2 stalk. Upon receptor-binding by the S1 subunit, an irreversible conformational switch occurs from the pre-fusion to post-fusion state, allowing the S2 subunit to fuse viral and cellular membranes [43,48,50,51]. The S2 subunit contains two heptad repeats (HR), HR1 and HR2, which are in the form of extended α helices, as well as the fusion peptide [45,52,53]. It is the HR1 and HR2 sections of S2 that fuse the viral membranes and carry out this irreversible conformational change [48,54,55]. The resulting series of conformational changes enables the fusion peptide to insert into the host membrane, forming a pre-hairpin intermediate state [56]. Although receptor-binding initiates the conformational changes which drives virus-cell membrane fusion [48], additional factors in the entry pathway include pH acidification, temperature changes or proteolytic activation. IBV [57] and SARS-CoV [58], have been shown to utilise the pH-dependent endocytic pathway. SARS-CoV has also been shown to rely on the activity of host cell proteases, which cleave and activate the S glycoprotein [59]. SARS-CoV-2 research has also identified that temperature may influence the affinity of S glycoprotein–receptor interactions with SARS-CoV-2 binding affinity to ACE2 (angiotensin converting enzyme II) decreasing at higher than optimal temperatures [60].

Advancements in the structural understanding of the S glycoprotein came through the determination of the pre-fusion trimeric structure by Cryo-EM of both mouse hepatitis virus (MHV) and the human coronavirus HKU1 (hCoV-HKU1) [47,48,49,62]. Both structures highlighted the critical role of the interaction between the S1 and S2 trimers in the stabilisation of the S glycoprotein in its pre-fusion conformation [48,49,62]. Since then, numerous other pre-fusion coronavirus cryo-EM structures have been generated, including IBV [53], MERS-CoV (Middle Eastern Respiratory Syndrome Virus) [63], SARS-CoV [63] and SARS-CoV-2 [64]. To obtain cryo-EM structures in pre-fusion format, di-proline (2P) mutations, have been utilised to increase the stability of the constructs (Figure 4B). Pallesen et al. [65] have shown that 2P substitutions in the loop between the HR1 and the central helix inhibits early triggering of the fusion protein and often increases expression yields of pre-fusion conformation ectodomains [65]. Subsequently, the introduction of two consecutive proline residues at the beginning of the central helix appears to be a generalisable method for retaining prototypical prefusion conformation of coronavirus S proteins. This approach has recently been utilised in the generation of COVID-19 vaccines produced by both Pfizer/BioNTech (BNT162b2) and Moderna (mRNA-1273). The transitional bend between HR1 and the central helix is fixed with 2P substitutions which stabilises the S protein at the prefusion state, which is key for vaccine development [47,64,65,66]. Whilst obtaining cryo-EM structures stabilised in pre-fusion conformation produces a higher yield, it is possible to obtain structures in post-fusion conformations with the structures for both MHV [48] and SARS-CoV [51] being solved and more recently that of SARS-CoV-2 [51].

## 4. The Spike Glycoprotein: Receptor-Binding Domains

**Figure 5:**
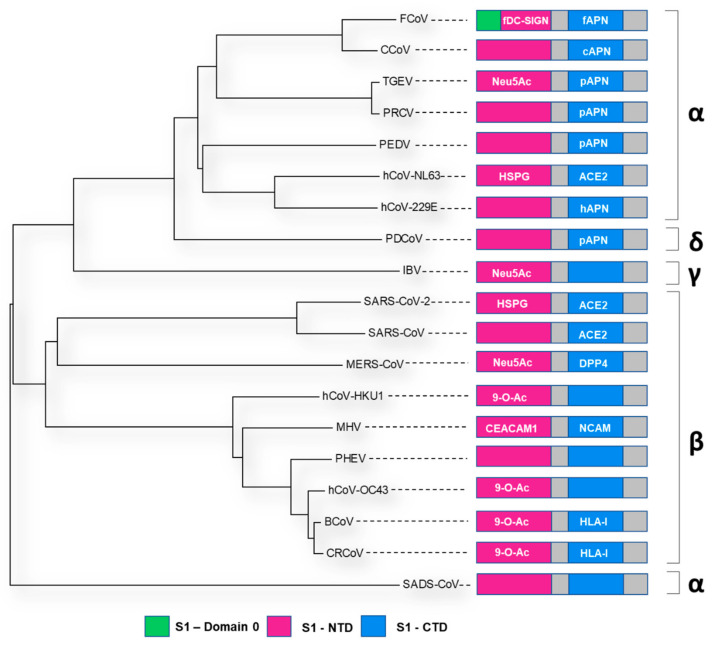
A midpoint rooted cladogram of representative coronavirus S glycoprotein sequences across the *alpha*-, *beta*-, *gamma*- and *delta*-genres. Representative strains from Genbank were used (Accesion Numbers outlined in Table 1). MUSCLE [22] was used to align the sequences in MEGA X [23]. A Neighbour-Joining cladogram was generated and the coronavirus genera denoted by Greek symbol (*alpha*-α, *beta*-β, *delta*-δ, *gamma*-γ). A linear schematic of the S glycoprotein S1 domain is also indicated for each virus (NTD in pink and CTD in blue—additional binding domain A of FCoV indicated in green). The receptor bound by the relevant domain is annotated accordingly.

**Figure 6:**
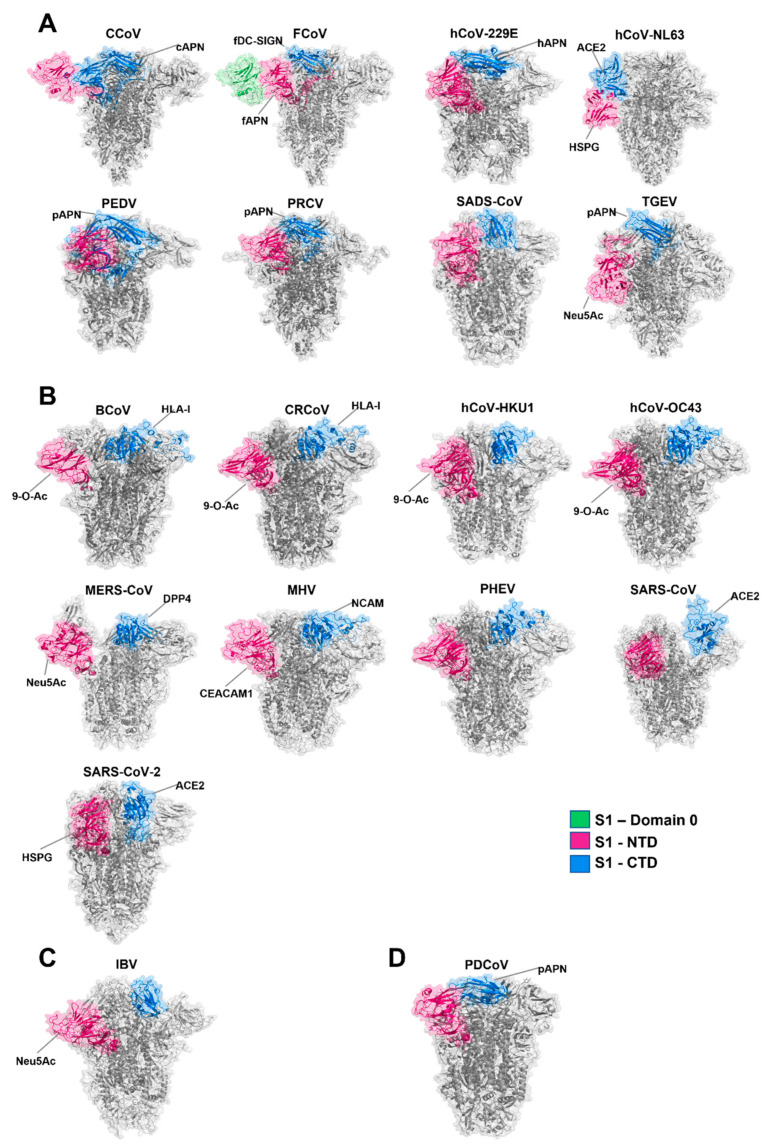
Spike glycoprotein schematic diagrams with putative receptor-binding domains indicated (as per Figure 5, NTD in pink and CTD in blue—additional binding domain A of FCoV indicated in green). (**A**) = *alphacoronavirus*, (**B**) = *betacoronavirus*, (**C**) = *deltacoronavirus*, (**D**) = *gammacoronavirus* genres. The Genbank accession numbers for the representative sequences used for modelling in PyMol [61], and the **RCSB PDB ID** used as a SWISS-MODEL [82] backbone are denoted in Table 1.

The S1 subunit contains two prospective receptor-binding domains (RBD), one located at the N terminus denoted S1-NTD and the other located at the C terminus, S1-CTD (Figure 5 and Figure 6). It is believed that the S1-NTD is responsible for sugar and carbohydrate compound binding, whereas the S1-CTD primarily binds proteinaceous receptors [67]. Previous research has highlighted that the S glycoprotein contributes towards both tissue and cellular tropism as well as virulence and host range [68,69,70,71,72]. This has been extensively documented throughout all the coronavirus genres (reviewed by Hulswit et al. [73] and Belouzard et al. [46]). Variance in tissue or host tropism can be the consequence of small or large changes within both the NTD and CTD regions [73,74]. For example, a large deletion in the S glycoprotein of Transmissible Gastroenteritis Virus (TGEV) resulted in the emergence of Porcine Respiratory Coronavirus (PRCV) [75,76,77], and two mutations, K479N and S487T within the S1-CTD resulted in interspecies transmission of SARS-CoV from palm civets to humans [78,79]. Another example is the G142D S1-NTD mutation observed in SARS-CoV-2 Delta variant, which is linked with increased transmissibility and immune evasion [80,81].

**Table 1:** All viruses included in Phylogenetic trees (Figure 1 and Figure 5) with Accession Number indicated for the representative sequence used in the alignments. NTD amino acid residue regions, CTD amino acid residue regions are also denoted. N/A denotes that this domain is not present for that virus. “?” denotes that the information is unknown/unavailable in the current literature. Any other relevant binding domain information and the **RCSB PDB ID** used as a SWISS-MODEL [82] backbone to generate the spike glycoprotein schematic models in PyMol [61] for Figure 4, Figure 5 and Figure 6 are denoted.

Virus	Representative Strain	Accession	NTD	CTD	Additional Information	RCSB PDB ID	Reference
CCoV	CCoV-A76	AY436637.1	? → 286	328 → 721	D3 = RBD: 526 → 676	6JX7	[83,84]
FCoV	FIPV-UU4	MH292846.1	276 → 540	541 → 695	Domain 0: 1 → 275; Domain A = NTD, Domain B = CTD	6JX7	[85]
229E	229E/HK20-42	MT797634.1	48 → 268	297 → 434		6U7H	[86,87]
NL63	NL63/RPTEC/2004	JX504050.1	210 → 480	481 → 616	Domain 0: 1 → 178; Domain A = NTD, Domain B = CTD	5S7S	[49,88]
PEDV	PEDV-CV777	AF353511.1	20 → 324	253 → 638	Domain 0: 1 → 219; Domain A = NTD, Domain B = CTD	6U7K	[89]
PRCV	PRCV/ISU-1	DQ811787.1	N/A	283 → 426		6JX7	[90]
SADS-CoV	SADS-CoV/CN/GDWT/2017	AVM41569.1	17 → 252	273 → 400		6M39	[91]
TGEV	TGEV-Purdue P115	DQ811788.1	17 → 245	506 → 655		6JX7	[74]
BCoV	BCoV-ENT (98TXSF-110-ENT)	AF391541.1	15 → 294	326 → 540		6OHW	[92]
CRCoV	CRCoV-BJ232	KX432213.1	?	?		6OHW	[93]
HKU-1	HKU1/human/USA/HKU1-12/2010	KF686346.1	14 → 294	310 → 673		5I08	[92]
OC43	OC43/LRTI_238	KX344031.1	15 → 298	?		6OHW	[92]
MERS-CoV	HCoV-EMC/2012	NC_019843.3	18 → 351	367 → 588	RBM: 484 → 567	5X5F	[94]
MHV	MHV-JHM.IA	FJ647226.1	15 → 296	326 → 567		3JCL	[95]
PHEV	PHEV-CC14	MF083115.1	15 → 300	311 → 608		6NZK	[92]
SARS-CoV	SARS-CoV/Tor2	NC_004718.3	13 → 318	323 → 502		5X5B	[96]
SARS-CoV-2	SARS-CoV-2/Wuhan-Hu-1	NC_045512.2	27 → 300	336 → 516		6VXX	[97]
PDCoV	PDCoV/USA/Ohio137/2014	KJ601780.1	52 → 277	302 → 422		6B7N	[98]
IBV	IBV/M41-CK	MK728875.1	21 → 237	269 → 414		6CV0	[53]

## 5. The Spike Glycoprotein: Cleavage

A significant distinction between the S glycoproteins of coronaviruses is whether they are cleaved or not during viral assembly and virion exocytosis [46]. Cleavage is a crucial factor in the final step of viral entry. With some exceptions, in most *alphacoronaviruses* and the *betacoronaviruses* SARS-CoV and SARS-CoV-2, the virions harbour a S glycoprotein that is uncleaved, whereas in some *betacoronavirus* including hCoV-OC43 [99] and all *gammacoronaviruses* including IBV, the protein is found cleaved. Cleavage typically occurs between the between the S1 and S2 subunits at what is referred to as the S1/S2 site, typically by furin, a Golgi-resident host protease; note that the subunits remain non-covalently linked [46,100]. Haan et al. [99] have shown that some alphacoronavirus S glycoproteins carry a furin enzyme recognition motif (RXXR) responsible for this cleavage. Interestingly, this furin enzyme recognition motif can be lost during cell culture adaptation by a single mutation within the cleavage motif; this, however, then preserves a heparan sulfate binding motif and renders infection by the virus heparan sulfate dependent [99]. This has been demonstrated for both FCoV and hCoV-OC43 [99].

Study of the SARS-CoV S glycoprotein has showed that cleavage at the S1/S2 site enhances fusogenicity of S glycoprotein, and therefore also increases the level of infectivity [45]. Surprisingly, SARS-CoV-2 unlike other *betacoronavirus* lineage B viruses, harbours a unique S1/S2 furin-recognition site, indicating that its S glycoprotein might possess some unique infectious properties [97,101,102]. The presence of a polybasic cleavage site (PBCS) in the SARS-CoV-2 S glycoprotein at the S1/S2 site has been proposed to act as factor in increased transmissibility of SARS-CoV-2 compared to SARS-CoV [103]. It is thought to do this by facilitating S glycoprotein precursor maturation by furin-like proteases in the producer cells rather than by endosomal cathepsins in target cells. In avian influenza viruses (AIVs), the polybasic cleavage site is a proteolytic excision site used by cellular proteases to activate a wide range of precursor proteins [104]. Multiple other proteases are reported to be responsible for cleavage, including TMPRSS2 [105,106]; cathepsin CTSL [107,108], and trypsin [109,110]. The acquisition of a polybasic cleavage site in the AIV surface glycoprotein haemagglutinin (HA), is a key feature of high pathogenesis [19,111,112] and virulence. However, although the SARS-CoV-2 S contains a polybasic cleavage site at the S1/S2 boundary, it was reported that furin cleavage of the S glycoprotein did not enhance SARS-CoV-2 entry into cells and in fact attenuates SARS-CoV-2 pathogenesis [97,113], challenging the well-established concept on the role of a polybasic cleavage site motif [97,113].

A second cleavage site at the S2′ (fusion peptide and the C-terminal region of S2) was also identified [109]. The S2′ site is in close proximity to the S1/S2 site, and cleavage of either one or both of them can yield the separation of the two S glycoprotein subunits [114]. This exists for some coronaviruses, including the Beaudette strain of IBV [100] and SARS-CoV 2 [113]. The S2′ site has been linked to an increase in vitro tropism for the Beaudette strain of IBV [100]. The S glycoprotein of the MERS-CoV was also found to be effectively cleaved by furin [115] with both the S1/S2 and S2′ sites having the RXXR furin recognition motif.

## 6. The Spike Glycoprotein: Glycosylation

The S glycoprotein is decorated with N-linked glycans, as demonstrated in studies using viruses belonging to the *Alpha*-, *Beta*- and *Gammacoronavirus* families [53,86,116]. The number of N-linked glycosylation sites varies between coronaviruses, with most IBV strains exhibiting 30–35 sites, compared to 60 in the S glycoprotein of SARS-CoV-2 [117,118]. *Betacoronavirus* S glycoproteins have also been shown to exhibit O- as well as N-linked glycosylation [119]. The interaction of glycans and host receptors has been studied in IBV, where a link was presented between glycosylation and lectin-mediated virus entry [120]. Glycosylation has also been shown to impact binding to sialic acids, where specific mutations to glycosylation sites in the IBV S glycoprotein abolished binding to sialic acids [121]. Recent studies using S glycoproteins expressed in insect and mammalian cells have revealed differences in the composition of the glycan profile, which has, in turn, been shown to impact interactions with host receptors. For example, differences in expression of different glycan types (complex vs. oligomannose) between SARS-CoV-2 variants was shown to alter the strength of S glycoprotein binding to ACE2 [122]. As well as affecting receptor interactions, mutations in S glycoprotein glycosylation sites have also been demonstrated to reduce infectivity and alter antigenicity in SARS-CoV-2 [123], highlighting the range of viral processes affected by these post-translational modifications.

## 7. The Haemagglutinin-Esterase Glycoprotein

**Figure 7:**
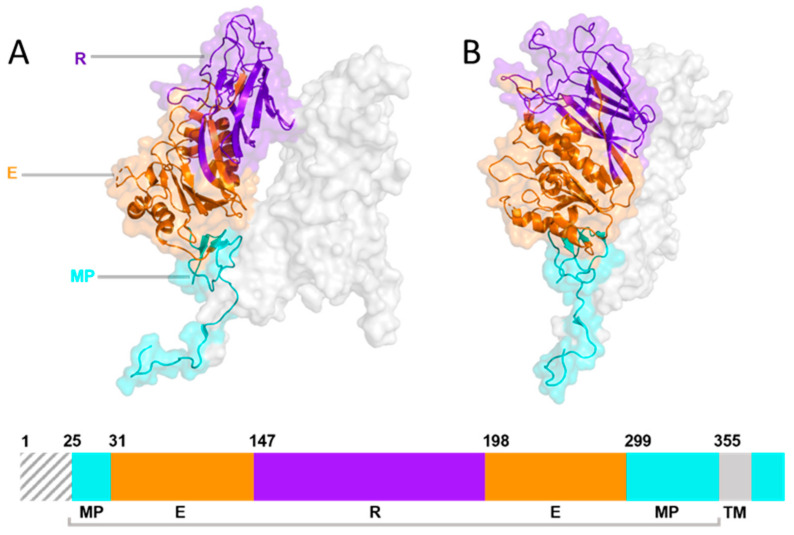
Structure and linear schematic of HKU-1 HE glycoprotein structure modelled in PyMol [61] using the Cryo-EM structure of HKU-1 [128] (**RCSB PDB**: 6Y3Y). (**A**) A single monomer is highlighted with each region of the HE glycoprotein annotated. (**B**) Linear schematic diagram of HKU-1 HE ectodomain.

**Figure 8:**
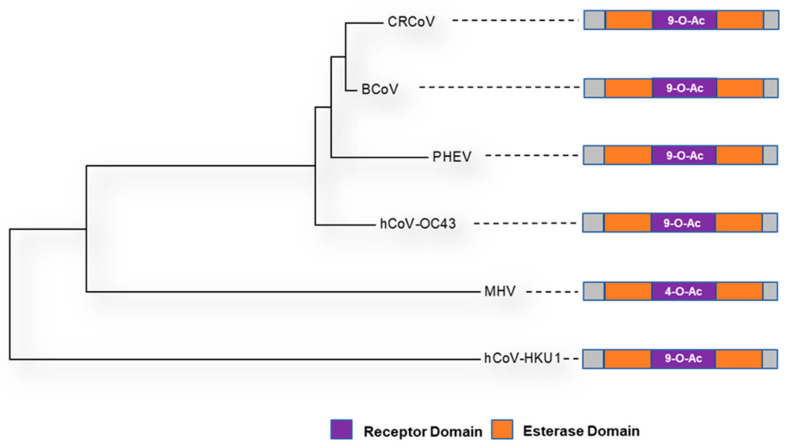
A midpoint rooted cladogram of betacoronavirus Lineage A viruses. Representative strains from Genbank were used (Accession Numbers outlined in Table 2). MUSCLE [22] was used to align the sequences in MEGA X [23]. A Neighbour-Joining cladogram was generated. A linear schematic of the HE glycoprotein lectin and esterase domains is also indicated for each virus (Esterase in orange and Lectin in purple). The receptor bound by the relevant domain is annotated accordingly.

**Figure 9:**
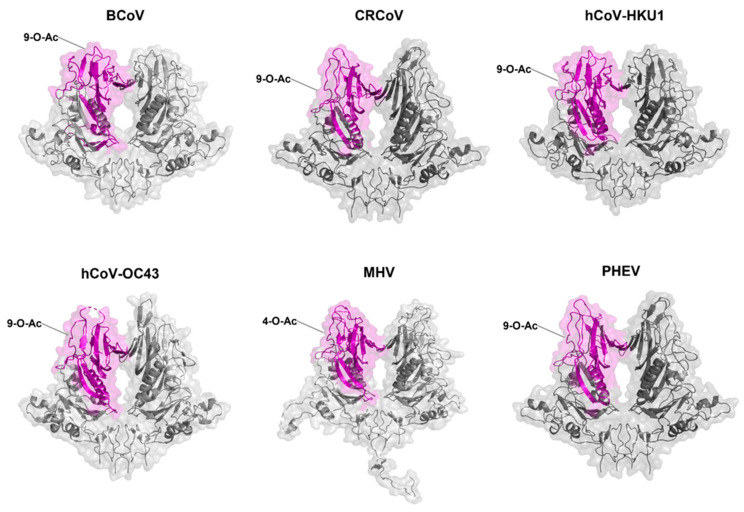
HE glycoprotein schematic diagrams with putative lectin binding domains indicated (as per Figure 8, Lectin domain in purple). The Genbank accession numbers for the representative sequences used for modelling in PyMol [61], and the **RCSB PDB ID** used as a SWISS-MODEL [82] backbone are denoted in Table 2.

Lineage A betacoronaviruses, including mouse hepatitis virus (MHV) and bovine coronavirus (BCoV), differ from other coronaviruses as their virions possess two types of surface proteins (Figure 2), both of which play key roles in attachment and receptor-binding [92]. In addition to the S glycoprotein, they also encompass 8-nm protrusions, unique to this clade of viruses, comprised of the homodimer hemagglutinin-esterase (HE). The HE protein is multi-functional, with orthologs embedded in the envelope of several viruses, not just coronaviruses, including both toroviruses and Influenza C viruses [36,124]. HE monomers have a bimodular structure with a carbohydrate-binding (lectin—R) domain attached to an enzymatically active sialate-O-acetylesterase (esterase—E) domain and a membrane-proximal domain (MP) [124,125,126] (Figure 7). HE, acts as a receptor-destroying enzyme (RDE) due to the presence of an appended 9-O-acetylated sialic acid-specific lectin domain (Figure 8 and Figure 9) [127]. The esterase region of the HE protein is responsible for the destruction of the receptor, the same action that is displayed by the neuraminidase protein in influenza A and B viruses [126]. Zeng et al. [126] have shown that the CoV HE arose from an influenza C-like HE fusion protein (HEF). The HE was transformed from a trimer into a dimer, with remnants of the fusion domain adapted to establish novel monomer–monomer contacts [126].

During pre-attachment, the RDE activity of HE prevents irreversible binding of virions to the decoy receptors that are universally found in the extracellular environment. At the end of the replication cycle, HE-mediated breakdown of cell-surface and intracellular receptors enables the release of viral progeny from the infected cell [129]. This has been observed in the viral replication of several lineage A betacoronaviruses including BCoV and MHV as well as Influenza C [126]. Interestingly, the HEs of two different human coronavirus strains, hCoV-OC43 [92,129] and hCoV-HKU-1 [92], which typically cause mild upper respiratory disease with symptoms of the common cold, have lost the ability to bind 9-O-acetylated sialic acids—the lectin domain has been rendered inactive for reason that still remain unknown; yet it remains functional in all other HEs studied so far [130]. Bakkers et al. [130] have demonstrated that the loss of the lectin affinity of the hCoV-OC43 lectin domain is due to a combination of four mutations—T114N, R177P, E178Q and F247L [130].

**Table 2:** All viruses included in the Phylogenetic tree (Figure 8) with Accession Number indicated for the representative sequence used in the alignments. Lectin Binding Domain (LBD) amino acid residue regions are also denoted. “?” denotes that the information is unknown/unavailable in the current literature. Any other relevant binding domain information, and the **RCSB PDB ID** used as a SWISS-MODEL [82] backbone to generate the HE glycoprotein schematic models in PyMol [61] for Figure 9 are denoted.

Abbreviation	Representative Strain	Accession	LBD	RCSB PDB ID	Reference
BCoV	BCoV-ENT (98TXSF-110-ENT)	AAK83355.1	141 → 283	3CL4	[131]
CRCoV	CRCoV-BJ232	AQT26497.1	?	3CL4	[132]
HKU-1	HKU1/human/USA/HKU1-12/2010	AGW27880.1	248 → ?	6Y3Y	[128]
OC43	OC43/LRTI_238	AOL02452.1	112 → 281	5N11	[129]
MHV	MHV-JHM.IA	AOL02452.1	146 → 298	4C7W	[131]
PHEV	PHEV-CC14	AVV64334.1	?	3I1L	[133]

## 8. Entry Receptors, Attachment Factors, and Cofactors: Protein Receptors

### 8.1. Angiotensin-Converting Enzyme 2 (ACE-2)

**Figure 10:**
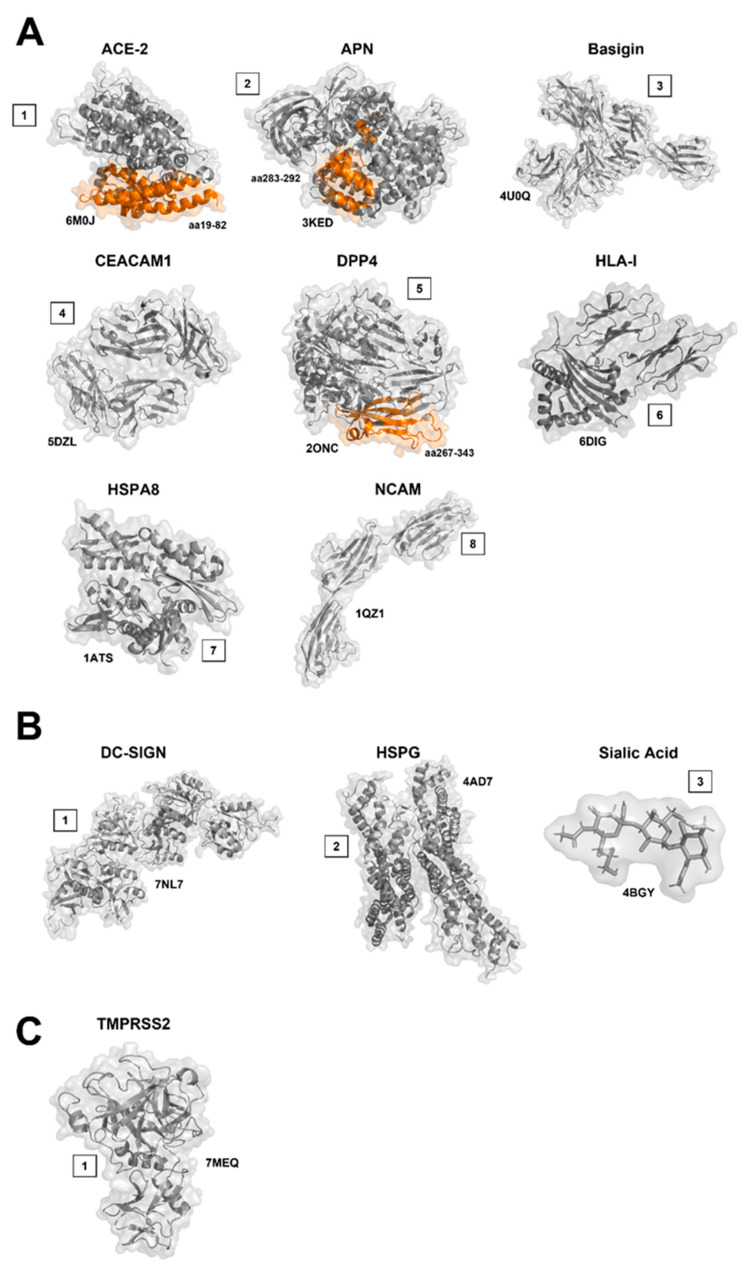
Receptor schematic diagrams with putative binding regions (where known) highlighted in orange, with amino acid residue locations noted. (**A**) = *Protein Receptors*, (**B**) = *Sugar Receptors*, (**C**) = *Other Binding Factors*. The RCSB PDB for the representative models made in PyMol [61] are denoted below the relevant model.

Angiotensin-converting enzyme 2 (ACE-2) (Figure 10A1), is a zinc metalloenzyme and carboxypeptidase [134] which is found attached to the cell membranes of cells located in the lungs, arteries, heart, kidney, and intestines of multiple different species including swine, cattle and humans [135,136]. It was first discovered as a homologue of ACE (Angiotensin-converting enzyme) and acts as its physiological counterbalance providing homeostatic regulation of circulating angiotensin II (Ang II) levels [134]. ACE-2 has many functions and though its primary substrate appears to be Ang II, it can hydrolyse a number of other physiological substrates [134,136].

Human ACE 2 (hACE 2) receptor-binding has perhaps been most extensively studied with regards to SARS-CoV [67,79,137]. Less than a year after the emergence of SARS-CoV, it was reported that ACE-2 was the cellular receptor [137,138]. Proteomic analysis revealed ACE-2 to be a high-affinity binding partner of SARS-CoV S1 and furthermore, inhibition of SARS-CoV infection of susceptible cells using antibodies against ACE-2, indicated that the protein facilitated SARS-CoV infection [138]. A second group independently identified ACE-2 as the receptor [139] by expression cloning of S glycoprotein fragments, and found that N-terminus 14–502 residues were sufficient to bind Vero E6 cells and act as the SARS-CoV RBD [138]. The RBD constantly switches between a standing-up position for receptor-binding and a lying-down position for immune evasion [63,140]. The binding affinity of the SARS RBD to different species of ACE-2 can be altered by altering specific residues (specifically K479N and S487T) which allowed cross-species transmission from palm civets to humans in a naturally occurring transmission event [78].

Unsurprisingly considering the 77% amino acid similarly of the S glycoprotein with SARS-CoV, SARS-CoV-2, also recognises hACE2 as its receptor [141,142,143]. The SARS-CoV-2 S glycoprotein has been shown to have broad tropism for other mammalian ACE-2 proteins including bovine, feline and canine [144]. The recently determined the crystal structure of SARS-CoV-2 RBD complexed with hACE2, revealed slight but functionally crucial differences between SARS-CoV-2 and SARS-CoV S glycoprotein in receptor recognition [29]. These differences allow for significantly higher hACE2 binding affinity for the SARS-CoV-2 RBD than the SARS-CoV RBD [29]. However, the cryo-electron microscopy (cryo-EM) structure of SARS-CoV-2 spike revealed that its RBD is mostly in the lying-down state [64,97], a state linked with ineffective receptor-binding and immune evasion. There have been conflicting reports on the hACE2-binding affinities of SARS-CoV-2 and SARS-CoV spikes [64,105,108]. A review by Harvey et al. [145] has identified key S glycoprotein mutations which affect virus neutralisation and variance in binding affinity to ACE-2 proteins.

Perhaps the most interesting research on hACE-2 binding is that it is also utilised by Human coronavirus NL63 (HCoV-NL63), an *alphacoronavirus* that was first identified in 2004 in a 7-month-old patient with a respiratory tract infection in the nasopharyngeal aspirate [8,146]. HCoV-NL63, unlike SARS-CoV and SARS-CoV 2, typically causes mild or subclinical infection. Infection of young children and immunocompromised adults can in some cases result in acute respiratory disease [147]. A minimal receptor-binding domain (RBD) that consisted of 141 residues (amino acids 476–616) was identified on the S1-CTD. The data suggests that the S1-CTD binding domain bound hACE-2 more efficiently than the full-length S glycoprotein and had a binding efficiency comparable to SARS-CoV. The crystal structure of NL63 S glycoprotein receptor-binding domain (RBD) complexed with human ACE-2 was generated [88] and identified three discontinuous receptor-binding motifs (RBMs) for ACE-2 binding [88]. Directed expression of the ACE-2 renders cells permissive to HCoV-NL63 infection, however, the presence of the receptor protein does not appear to directly correlate with the adhesion of virions to the cell surface, suggesting that another attachment factor is required [146].

### 8.2. Aminopeptidase N (APN, CD13)

Aminopeptidase N (APN) (Figure 10A2) is a type II metalloprotease belonging to the M1 family of the MA clan of peptidases [148]. It consists of 967 amino acids with a short N-terminal cytoplasmic domain, a single transmembrane region, and a large cellular ectodomain which contains the active site [149]. APN exists in two forms, membrane APN and soluble APN. It is present in a wide variety of human organs, tissues and cell types and is multifunctional with numerous roles in several physiological processes. It is a zinc-dependent aminopeptidase which cleaves one residue from the N terminus of many physiological peptides [150,151]. Furthermore, it also serves as an entry receptor for coronaviruses and other human viruses [150]. Sequence comparisons with known enzymes of this class showed that CD13 (cluster of differentiation 13) and aminopeptidase N are identical [149]. The crystal structure for both porcine and human APN has been determined [150,152].

APN is the most widely studied protein receptor in the veterinary field of coronavirus research, with the receptor, specifically porcine APN (pAPN) first identified for TGEV [153]. TGEV is an enteropathogenic *alphacoronavirus* that causes diarrhoea in pigs [154]. Binding activity to APN is required for TGEV to initiate cellular infection [153] and the binding region of pAPN is believed to be AA residues 522–744 of the S glycoprotein [155,156,157]. Interestingly PRCV, an S gene deletion mutant of TGEV which displays altered in vivo tropism [90,158,159] binds pAPN comparably to TGEV [74]. The altered in vivo tropism exhibited by PRCV is therefore likely not the result of differences in pAPN binding.

Two further porcine coronavirus are reported to utilise pAPN including Porcine epidemic diarrhoea virus (PEDV), first isolated in 1977 [160] and Porcine Deltacoronavirus (PDCoV), initially detected in 2009, but its etiologic role was not identified until 2014 [98,161,162]. Several studies have shown that pAPN acts as the primary PEDV receptor [163,164,165] with the minimal binding region identified to be located within residues 25–88 [166]. To date, however, there is no conclusive data concerning the exact location of the PEDV RBD and the key amino acids that participate in receptor-binding. It has however been demonstrated that a recombinant PEDV fragment (S1-NTD-CTD—the domains overlap) can bind both pAPN and hAPN efficiently (≥75% sequence identity) [164].

Interestingly PDCoV, which belongs to the *Deltacoronavirus* genus that comprises predominantly avian coronaviruses, utilises a conserved region of APN and is able to infect cell lines derived from multiple species, including humans, pigs, and chickens [89,167]. Transient expression of porcine, feline, human, and chicken APN renders previously non permissive cells susceptible [89,168] and phylogenetic analysis suggests PDCoV evolved relatively recently from a host-switching event between birds and mammals [162,167]. Binding of PDCoV to an interspecies conserved site on APN may have facilitated this species barrier jump [89]. It has been reported that PDCoV interacts with APN via domain B (S1-CTD) of its S glycoprotein. Wang et al. [169] found that the soluble S1 protein of PDCoV bound to the surface of target porcine cell lines known to express pAPN and that the PDCoV-S1 interacted with pAPN by coimmunoprecipitation in pAPN cDNA-transfected cells and by dot blot hybridisation assay [169]. PDCoV-S1 appeared to have a lower pAPN-binding affinity and likely consequent lower infection efficiency in pAPN-expressing refractory cells than TGEV-S1, which binds pAPN on the same domain, suggesting that there may be differences between these two viruses in the virus-binding regions on pAPN [169].

Canine coronavirus (CCoV) and feline coronavirus (FCoV) have also been shown to utilise APN as a receptor [170]; canine APN (cAPN) and feline APN (fAPN) respectively. Reassortant FCoVs gave rise to mutant viruses which lead to the development of feline infectious peritonitis (FIP). Both CCoV and FIPV are divided into two genotypes: I and II. These genotypes are then further divided into two subtypes, IIa and IIb [171]. In type IIb CCoV, the 5′-terminal region of the S gene is similar to that of TGEV, and it is thought to have emerged through recombination of both type IIa CCoV and TGEV [171,172]. Type II FIPVs also display close antigenic links to TGEV [173]. Though serotype I CCoV/FIPV have an ill-defined receptor, both Serotype II CCoV/FIPVs utilise APN as a receptor [84,174]. Within the serotype II viruses, variant CoVs have also been identified where the N-terminal domain of the S glycoprotein is highly homologous to either TGEV or to serotype I CCoV/FIPVs [170]. These variant viruses are suggested to have major antigenic differences when compared to prototype serotype II CoVs [172]. Genomic analysis of several CCoV strains shows that variant CCoV-A76 possesses a distinct spike; the result of recombination between type I and type II CCoV, that occurred between the S1-NTD and CTD [84]. This data suggests that CCoV-A76 represents a recombinant coronavirus form, with distinct host cell tropism and potentially novel receptor recognition [84].

To date, only one human coronavirus has been shown to use APN as a receptor [87,175]. HCoV-229E was first isolated in the mid-1960s from a person with a common cold [176,177]. The hAPN RBD lies between amino acids 417 and 547 in the S1-NTD [178]. HCoV-229E can utilise either hAPN or feline APN (fAPN) as a receptor [179,180], but there is no detectable binding with pAPN [153,181]. Kolb et al. [181] identified a region of the hAPN from amino acid residues 288 to 295 to be essential for HCoV-229E infection [181]. Recent studies have shown that that the 229E receptor activity with hAPN can be abolished by the addition of a single N-linked glycosylation site at amino acid 291 of hAPN, corresponding to a naturally occurring N-glycosylation site on pAPN [182].

IBV is the aetiological agent of infectious bronchitis (IB); an economically important and highly transmissible respiratory disease of poultry [183,184,185,186,187]. Although the primary receptor for IBV cellular entry remains elusive, APN has been suggested. Transient transfection with fAPN plasmids, enabled infection into previously non permissive BHK-21 cells [188]. However, later research has indicated that fAPN is actually not a functional receptor with low entry efficiency detected in BHK-21 cells following fAPN transient transfection and constitutive expression [189]. Due to variance between different species of APN and non-relevant cell types, these results make the role of APN in IBV infection unclear. IBV has however been shown to proliferate in non-permissive HeLa cells transfected with recombinant Galliforme APN (gAPN) plasmids [190], suggesting that IBV could bind to both the native form and prokaryotic expressed versions of gAPN proteins.

### 8.3. Basigin (BSG/CD147/EMMPRIN)

Basigin (Figure 10A3), also known as CD147 or EMMPRIN, is a transmembrane glycoprotein belonging to the Ig superfamily [191]. It is involved in tumour development, plasmodium invasion and viral infection [192,193]. Basigin plays a functional role in facilitating SARS-CoV invasion in host cells, and CD147-antagonistic peptide-9 has an inhibitory effect on SARS-CoV [192], reaffirming the importance of CD147 in virus invasion for host cells. Additionally, Meplazumab, a humanised anti-CD147 antibody, could effectively inhibit the viruses from invading host cells by blocking CD147 [191].

### 8.4. Carcinoembryonic Antigen-Related Cell Adhesion Molecule 1 (CEACAM1)

Carcinoembryonic antigen-related cell adhesion molecule 1 (CEACAM1) (Figure 10A4) is a human, biliary glycoprotein, also known as CD66a (Cluster of Differentiation 66a) and is a member of the carcinoembryonic antigen (CEA) gene family. CEACAM1 is expressed on various epithelial cells, endothelial cells, and hemopoietic cells [194,195]. It functions as a cell adhesion molecule [195,196,197,198], a signalling molecule [199], and an angiogenic factor [200] and is classified in the immunoglobulin (Ig) superfamily. It has two different functions for viral entry into cells: binding the S glycoprotein and activating the S glycoprotein to execute virus-cell membrane fusion. CEACAM1 is composed of four Ig-like ectodomains (in the order N, A1, B, and A2 or D1, D2, D3, and D4 from the N terminus), a transmembrane domain (TM), and a cytoplasmic tail [21,198]. CEACAM1 is also found in other animal species including mice [196].

Mouse hepatitis coronavirus (MHV) was first discovered in 1949 and is the most studied coronavirus in animals [201], acting as a model organism for studying coronaviruses [202]. MHV uses the N-terminal domain (NTD) of its S glycoprotein as its receptor-binding domain [203]. The host cell receptor used by murine coronaviruses is generally CEACAM1 (mCEACAM1), which is unusual in that the S1-NTD usually binds to sugar molecules rather than exclusively protein-protein interactions [203]. Although receptor-binding is usually vital in cellular entry, a phenomenon described as ‘receptor-independent spread’, was shown by the MHV-JHM strain, meaning it can spread from infected mouse cells to cells lacking mCEACAM1a [204]. The MHV-JHM strain could potentially use an alternative, less effective, unknown receptor to initiate infection [205]. Once primary infection is established in the murine host glial cells, the JHM strain was shown to rapidly spread via cell–cell fusion and syncytia formation in a receptor-independent manner [206].

### 8.5. Dipeptidyl-Peptidase 4 (DPP4)

Dipeptidyl-peptidase 4 (DPP4) (Figure 10A5), also known as CD26 (cluster of differentiation 26) is a protein that, in humans, is encoded by the DPP4 gene [207]. It is found on the surface of cells in the airways, including the lungs as well as in the kidneys. DPP4 is a serine exoprotease that cleaves two residues from the N terminus of many physiological peptides [208,209,210]. DPP4 is also known to cleave a broad range of substrates which in the majority of cases lose their biological activity often leading to a shift in the receptor subtype binding [211]. MERS-CoV which causes severe pulmonary disease in humans [212,213] utilises DPP4 as an entry receptor [214]. Receptor-binding was attributed to the S1-CTD AA residues 367–606, which was consequently denoted the RBD [215].

### 8.6. Human Leukocyte Antigen I (HLA-I)

The human leukocyte antigen (HLA) (Figure 10A6) system is a complex of genes on chromosome 6 in humans which encode cell-surface proteins responsible for the regulation of the immune system [216]. The HLA system is also referred to as the human equivalent of the major histocompatibility complex (MHC) found in many animal species [217]. MHC class I proteins form a functional receptor on most nucleated cells [217].

HLA molecule have been identified as a potential receptor for both BCoV and Canine Respiratory Coronavirus (CRCoV). The interaction between the BCoV and HLA-I molecules in vitro using HRT-18G cells was blocked using polyclonal antibodies, preventing subsequent infection by BCoV [218]. Additionally, saturation with HLA-I was shown to block HRT-18G cellular infection of CRCoV [218]. HLA-1 was also previously reported to facilitate entry of both OC43 and HKU-1 into cells, however when investigated, HLA-1 did not affect replication of OC43 in HRT-18G, suggesting that it is not an entry receptor for this virus [218].

### 8.7. Heat Shock Proteins (HSPs)

Heat shock proteins (HSP) (Figure 10A7) are a family of proteins that are produced by cells in response to exposure to stressful conditions [219,220]. They are found in virtually all living organisms, from bacteria to humans and are named according to their molecular weight; for example, HSP60 in 60 kilodaltons (kDa) in size, HSP70 is 70kDa and Hsp90 is 90 kDa respectively [221]. Many HSPs perform chaperone functions by stabilising new proteins to ensure correct folding or helping to refold proteins that were damaged by the cell stress [222]. HSP70 has been shown to localize at or near the surface of plasma membranes of cells [223]. Whilst the role of HSPs as entry and/or binding have not been widely studied, certain coronaviruses including IBV, MERS-CoV, SARS-CoV and SARS-CoV 2 can utilise HSPs in this way.

Infectious bronchitis virus (IBV) has been reported to use HSPs as attachment factors. Specifically, HSP Member 8 (HSPA8) [224], HSP47 [225] and HSP70 [226]. HSP47 was found to interact specifically with the IBV S1 protein [225], after a chicken kidney cDNA library was screened using a yeast two-hybrid system assay. Expression of the S1 subunit and recombinant HSP47 in HeLa cells demonstrated colocalization. Amino acids 340–470 in the S1 subunit were critical for the interaction [225]. For HSPA8, in vitro assays showed that recombinant protein HSPA8 and anti-HSPA8 antibody could inhibit IBV M41 infection of chicken embryonic kidney cells [224]. HSPA8 was shown to interact with the S1-NTD of IBV strains, M41, Beaudette, H120 and QX [224]. HSPA8 is a member of HSP70 family and is also referred to as HSP71. HSPA1, also known as HSP72, is also reported to interact with the IBV-S1 RBD [226]. Recombinant S1-NTD proteins of M41 and SCZJ3 were expressed, and the binding capacities to chicken tissues investigated. Protein histochemistry showed that both proteins could bind to lung and kidney tissue, and that SCZJ3 displayed a distinctive staining pattern in the proventriculus [226]. Affinity chromatography assay detected a 70 kDa band corresponding to HSP70. Infection of chicken embryo kidney cells by SCZJ3 was found to be inhibited by anti-HSP70, indicating that HSP70 is part of the receptor complex of IBV [226]. The inhibitory data towards M41 was not reported [226].

HSP90 has been observed as a host dependency factor for several human coronaviruses including MERS-CoV, SARS-CoV and SARS-CoV-2 [15]. In mammalian cells, there are two cytosolic isoforms of HSP90, the stress-inducible HSP90α and constitutively expressed HSP90β [227]. Li et al. inspected the role of HSP90 for coronavirus propagation. They found that the HSP90 inhibitor, 17-AAG, significantly reduced MERS-CoV propagation in physiologically-relevant human intestinal organoids and cell lines. They also found that, siRNA depletion of HSP90β, but not HSP90α, significantly restricted MERS-CoV replication. Additionally, they demonstrated that 17-AAG substantially inhibited the replication of SARS-CoV and SARS-CoV-2, indicating that HSP90 interacts with multiple human coronaviruses [227]. They also proposed that HSP90 inhibitors can be repurposed as a potent and broad-spectrum antiviral against human coronaviruses [227].

### 8.8. Neural Cell Adhesion Molecule (NCAM)

Neural cell adhesion molecule (NCAM) (Figure 10A8), also known as CD56, is a homophilic binding glycoprotein expressed on the surface of neurons. It is part of the Ig superfamily. NCAM has been implicated as having a role in cell–cell adhesion [228].

Porcine hemagglutinating encephalomyelitis virus (PHEV), a *Betacoronavirus* that causes encephalomyelitis in piglets younger than 3 weeks [229,230]. PHEV is a highly neurovirulent virus that spreads to the central nervous system via peripheral nerves [231], where nerve cells are a target for viral replication; however, the mechanism by which PHEV enters nerve cells is not known. The neural cell adhesion molecule (NCAM, also known as CD56) is a homophilic glycoprotein expressed on the surface of nerve cells. It has been demonstrated that NCAM participates in the process by which PHEV infects neurons and can act as a receptor [232]. To identify the crucial domain of the S1 that interacts with NCAM three truncated fusion proteins spanning the entire S1 subunit were screened using a GST pull-down experiment; the interactions were further confirmed by a yeast two-hybrid system assay. The results showed that the S fragment (amino acid residues 277–794) could interact with NCAM, and a smaller fragment (258-amino-acid fragment, residues 291–548) located within the S277-794 fragment may be the RBD of the PHEV S glycoprotein [229].

## 9. Entry Receptors, Attachment Factors and Co-Factors: Sugar Receptors

### 9.1. Dendritic Cell-Specific Intercellular Adhesion Molecule Grabbing Non-Integrin (DC-SIGN)

DC-SIGN (dendritic cell-Specific intercellular adhesion molecule grabbing non-integrin) (Figure 10B1) also known as CD209 (Cluster of Differentiation 209) is a protein encoded by the CD209 gene. DC-SIGN is a C-type lectin receptor present on the cell surface which recognises and binds with high affinity to high-mannose type N-glycans [233]. Besides functioning as an adhesion molecule, recent studies have indicated that DC-SIGN can initiate innate immune responses by modulating toll-like receptors, though the detailed mechanism is not yet known [234]. An additional receptor has also been identified to play a role in coronavirus receptor-binding, CD209L (also called L-SIGN, DC-SIGNR, and DC-SIGN2) [235].

Both serotype I and serotype II FIPVs use feline dendritic cell-specific intercellular adhesion molecule 3-grabbing nonintegrin (fDC-SIGN) as a coreceptor to recognise high-mannose glycans [236]. Domain A of FIPV S glycoprotein is densely decorated with high-mannose-type glycans, which could be involved in interacting with fDC-SIGN [85]. In vitro infection was strongly reduced by mannan, a competitive inhibitor of DC-SIGN binding; with this action circumvented through the addition of human DC-SIGN [237].

L-SIGN has also been identified as an alternative receptor for SARS-CoV; when transfected into Chinese hamster ovary cells, cells became susceptible to infection (Jeffers et al., 2004). Immunohistochemistry showed that L-SIGN is expressed in human lung in type II alveolar cells and endothelial cells, both potential targets for SARS-CoV [238]. Interestingly both DC-SIGN and L-SIGN can enhance infection of cells that co-express the receptor, ACE-2 [58,236,238]. SARS-CoV interaction with DC-SIGN was demonstrated using a soluble S-based binding assay in which DC-SIGN was transiently overexpressed in 293T cells. The S1 domain of SARS-CoV was found to be sufficient to mediate the interaction with DC-SIGN lectins [236]. It has been shown that DC-SIGN, can augment NL63 infection alongside its reliance on ACE-2 for viral entry [239]. Though the spike of both NL63 and 229E are highly conserved, DC-SIGN does not enhance viral infection of 229E, however L-SIGN expressed in non-susceptible cells can bind HCoV-229E, despite not utilising ACE2 as a receptor [240].

### 9.2. Heparan Sulfate (HS, HSPG)

Heparan sulfate proteoglycans (HSPGs) (Figure 10B2) encompass a diverse class of proteins defined by the inclusion of HS glycosaminoglycan (GAG) polysaccharide chains [241]. HS is a polymer of repeating N-acetyl glucosamine (GlcNAc)-d-glucuronic acid (GlcA) disaccharide units which is found in all animal tissues and cells [242]. It occurs as a proteoglycan (HSPG) in which two or three HS chains are attached in close proximity to extra cellular matrix or cell surface proteins [243,244] where they interact with numerous ligands [245]. HSPGs are highly conserved among both vertebrates and invertebrates and have multiple functions. They contribute to basal membrane organisation and mediate cell adhesion and motility. Specifically at the cell surface, HSPGs serve as endocytosis receptors and are also involved in the endocytosis of cellular receptors [246].

For both SARS-CoV and SARS-CoV-2, ACE-2-mediated entry requires the cell surface heparan sulfate (HS) as an assisting cofactor [143]. Entry of SARS-CoV pseudovirus can be inhibited by the removal of HSPGs via heparinase treatment [247]. SARS-CoV-2 infection has also been shown to be dependent on HS with in vitro treatment the competitive inhibitor heparin dose-dependently reducing SARS-CoV-2 pseudovirus infection [30]. Additionally, pulldown assays demonstrated that the purified S ectodomain readily bound to heparin-conjugated beads. Unsurprisingly given the shared use of ACE-2 receptor, HSPGs have been demonstrated to enhance HCoV NL63 infection [146]. HS binding has been clearly demonstrated for MHV, where infection by MHV-A59 strain (which is solely dependent on mCEACAM1a binding for entry), led to the emergence of variant strains with mutations and a short 7 amino acid insertion in the S1 subunit; _492_TQTTRTKKVPKPKS_505_ that introduces multibasic sites at different locations within the S glycoprotein [248,249]. These modifications allow HS mediated entry into cells. The 7 amino acid insertion identified in mutant viruses is located in the CTD, and yet allows for dual-binding competency to HS and mCEACAM1a as well as dependency on both factors for host cell entry [250]. Additionally, the added mutations introducing a multibasic HS-binding site within in the S2 subunit were found to remove the need for mCEACAM1a binding allowing for virus entry to be solely dependent on HS [205]. These mutations are specifically located at the S2′ cleavage site.

HS has also been identified as a selective attachment factor for IBV strain Beaudette (Mass serotype) [100,251]. Beaudette is an embryo-adapted virus strain with extended species tropism in cell culture [251,252] and was found to contain a recognised HS-binding site (between amino acid residues 686 and 691 of the S2 subunit of the Beaudette S glycoprotein), indicating that the Beaudette virus may use HS as a selective receptor [251]. While the S1 subunit of IBV contains the receptor-binding domain (S1-NTD) and is responsible for binding to host cells [253,254], it was determined that infectivity for Vero cells is mediated by the Beaudette S2 subunit, in particular, the Beaudette-specific motif _686_SRRKRSLIE_694_ surrounding the S2′ cleavage site [100]. An additional furin cleavage motif within the putative HS binding site was identified with a role in viral entry and syncytium formation in vitro [255]. Cleavage was mapped to arginine residue 690 [255].

### 9.3. Sialic Acid (SA)

Sialic acids (SA) (Figure 10B3) are a class of alpha-keto acid sugars with a nine-carbon backbone [256]. Sialic acids are commonly part of glycoproteins, glycolipids, or gangliosides, where they decorate the end of sugar chains at the surface of cells or soluble proteins [257]. The most frequently occurring member of the sialic acid family is N-acetylneuraminic acid, followed by N-glycolylneuraminic acid and O-acetylated derivatives, and up to now over about 80 neuraminic acid derivatives have been described [167]. The most common member of this group is N-acetylneuraminic acid (Neu5Ac or NANA) found in most animals and widely distributed in animal tissues [167,258,259,260].

SAs act as the primary receptor for several other viruses including Influenza viruses, adenoviruses and rotaviruses [261]. Sialic acid interactions are particularly well documented within the Influenza A virus field of research, with Neu5Ac documented as their primary receptor [262]. Among coronaviruses, several members have known interactions with sialic acids, including IBV and TGEV [157,263,264]. Human coronaviruses such as MERS-CoV have been shown to have interactions with SA. Cryo-EM structures of the MERS-CoV S in complex with 5-*N*-acetyl neuraminic acid, 5-*N*-glycolyl neuraminic acid, sialyl-Lewis^X^ (also known as CD15s), α2,3-sialyl-*N*-acetyl-lactosamine and α2,6-sialyl-*N*-acetyl-lactosamine were generated [265]. This data demonstrates that receptor recognition occurs via a conserved region that is essential for MERS-CoV S-mediated attachment to sialosides and subsequent entry into human airway epithelial cells [265]. The data also highlights that the MERS-CoV S glycoprotein sialoside specificity suggests preference for α2,3-linked over α2,6-linked receptors [265]–this is unusual for human respiratory viruses as α2,3-linked sialic acids are avian-like receptors, but are found in abundance in the lower respiratory tract [18], which would account for the receptor usage of DPP4 in the lung epithelial cells.

Whilst PEDV uses pAPN as a primary receptor, data generated by Pen et al. [266], using a dot blot hybridisation assay, demonstrated that the PEDV S1-NTD-CTD fragments also are capable of binding both bovine and porcine mucins, which contain a mixture of varying sugar types. Treatment of mucins with neuraminidase (to remove parts of the surface sugars), reduced the binding by PEDV S1-NTD-CTD, suggesting that sugar serves as a co-receptor for PEDV. Glycan screening identified Neu5Ac as the preferential sugar type for PEDV entry [164].

Data produced by Yang et al. [85] has defined 5 distinct domains on the FCoV S glycoprotein (within the S1 subunit): domain 0 (residues 1 to 275), domain A (residues 276 to 540), domain B (residues 541 to 695), domain C (residues 696 to 754), and domain D (residues 755 to 791). They employed a glycan array to test the lectin activities of the full-length S glycoprotein and 3 truncated variants, domain 0 only, domains 0 and A, and domains 0, A, and B [85]. Three groups of glycan structures were found to be recognised by all 4 variants; sialylated Galβ(1 → 4)Glcβ-core structures, sialylated Galβ(1 → 3)GalNAcβ-core structures, and oligo-glucose (Glc) structures [85]. The data suggests that positive recognition and binding by domain 0 prefer a minimum Galβ(1 → 3)GalNAcβ-core structure sialylated at the 6 position of GalNAc [85].

The JHM strain of MHV has also been shown to bind sialosides [267], with the JHM strain found to bind to SA. The sialic acid binding activity was mapped to the NTD (S1A) domain of MHV-JHM, which still retains its proteinaceous mCEACAM1a receptor-binding capability [205]. This illustrates the flexible nature of coronavirus S glycoproteins with an NTD capable of dual-binding modalities enabling attachment to both carbohydrate (sialic acids) and protein (mCEACAM1a) receptors [205]. The study suggests that MHV-JHM attachment likely occurs in a two-step fashion with low affinity binding to sialic acids followed by higher affinity binding to mCEACAM1a protein receptor [205]. MHV also harbours a HE protein [126]. It functions both as a lectin and a receptor destroying enzyme (RDE), thanks to its sialate-9-O-acetylesterase activity [126]. Notably, for MHV, binding to O-acetylated sialic acids was shown to be mediated solely by its HE protein and not S [131].

#### 9.3.1. Sialic Interactions with Both the Spike and Haemagglutinin-Esterase Glycoproteins

Whilst there is a SA binding domain on the S glycoprotein (S1-NTD), the interaction with SA can be mediated not solely by the S glycoprotein but also, in some cases, in those coronaviruses that encode it—the HE glycoprotein, as demonstrated by MHV [131].

HCoV-HKU1 [268] possesses both a S glycoprotein as well as a surface HE protein. The HE of HKU-1 has lost its ability to bind 9-O-Ac-Sia as the HE lectin domain has been rendered inactive [130]. The function of HKU1-HE remains largely undetermined [269]. Whilst it is known that HKU1 employs glycan-based receptors carrying 9-O-acetylated sialic acid (9-O-Ac-Sia), there is limited structural information on how the S glycoprotein of HKU1 binds ligands. HKU1 S was recently reported to bind to its receptor via a domain other than S1-NTD [270], and binding to 9-O-Ac-Sia was reportedly not detectable. However, pre-treatment of cells with neuraminidase and trypsin greatly reduced the binding, suggesting that the binding was mediated by sialic acids on glycoproteins [269]. HKU-1 is one of seven hCoVs identified to date and the only one with an unidentified cellular receptor [269], but it does exploit O-Ac-Sia as a cellular attachment receptor determinant to initiate the infection of host cells [269].

hCoV-OC43 similarly to HKU-1, also contains an HE protein [271,272,273]. Again, similarly to HKU-1, the HE of OC43 has lost its ability to bind 9-O-Ac-Sias [130]. The S glycoprotein of OC43 binds to O-acetylated sialic acid through domain A (S1A) on the S1-NTD, as demonstrated by in vitro binding assays (Peng et al., 2011b). The apo-structure of the BCoV S1A lectin domain was solved and believed to be highly similar to OC43. However attempts to solve the holo-structure reportedly failed [266]. Based on the galectin-like fold of the S1A domain and mutational analysis, the RBS was predicted [266]. Although this model remains to be confirmed, it has been widely accepted by the field [47,270]. Findings by Hulswit et al. indicate that the actual S1A RBD in the S glycoprotein could map elsewhere than currently believed [92]. They also propose that suggested site is not exclusive to just OC43, but in fact is also in the S1A domain of HKU1 [92]. Abi and Keha et al. [274] have identified that the R2-loop in the lectin domain acts in ligand binding, and amino acid substitutions within this domain could alter receptor-binding. They hypothesise that HCoV-OC43 may have evolved during adaptation by gradually losing its lectin activity due to AA mutations in the R2-loop of the lectin-binding domain in the HE protein [130,274]. Additionally, Szczepanski et al. [218] have indicated that the documented receptor interactions for OC43 may actually differ from the literature; they therefore conducted a series of experiments examining receptor usage. Haemagglutination assays indicated binding to mouse erythrocytes which are highly saturated with sialic acids. Neuraminidase treatment of cells also reduced viral attachment for OC43, however it did not affect the level of viral replication, suggesting that SAs do not facilitate entry of OC43. The SA could be utilised for anchoring the virus to the cell surface, or for binding as a decoy receptor. Examination of the SAs on the cell surface indicated a preference of binding to α2,6-linked, mammalian-like SA [218].

Although OC43 is an endemic respiratory pathogen, it originated from an independent zoonotic introduction, it is in fact closely related to bovine coronavirus (BCoV) [92]. The suggested bovine-to-human spillover of BCoV was proposed to have happened around the year 1890, based on the S gene sequences of BCoV and HCoV-OC43 [275,276,277,278]. BCoV initiates infection by attachment to cell surface receptors the crucial component of which is N-acetyl-9-O-acetylneuraminic acid [279]. Data by Schultze et al. [279] suggests that both glycoproteins and glycolipids can serve as receptors for BCoV provided they contain 9-0-acetylated sialic acid. They also suggest that following initial binding to sialic acid-containing receptors, the S-protein interacts with a specific protein receptor. This interaction may result in a conformational change that exposes a fusogenic domain and thus induces the fusion between the viral and the cellular membrane [279]. When assessing the S-protein sialic-acid binding capabilities, sialic acid lacking a 9-0-acetyl group was not effectively bound. The apo-structure of the BCoV S1^A^ lectin binding domain was solved but attempts to solve the holo-structure reportedly failed [266]. Whilst the HE of both OC43 and HKU-1 have lost their lectin activity [67], BCoV HE can still readily bind lectins, with preference for 9-O-Ac-Sia [274]. Studies by Abi et al. and Keha et al. [274] have noted that the R3-loop is composed of 13 aa (aa 207–219) in the BCoV HE, and residues 211–214 are essential for receptor-ligand interaction [126]; and that AA insertions between AA 211 and 212 could alter the spatial conformation of the receptor-binding site [280]. Szczepanski et al. [218] have suggested that similarly to OC43, previous BCoV receptor-binding observations indicate that the role(s) of these receptors may differ from those previously reported. Hemagglutination assays identified that BCoV also agglutinated mouse erythrocytes, which are rich in SAs [218]. To determine the importance of SAs for attachment, they treated cells with neuraminidase (NA) prior to infection and then examined viral attachment, showing a reduction in attachment, but similarly to OC43, no reduction in viral replication, suggesting again that SAs do not facilitate entry for BCoV. When examining the cell surface receptors they identified that yet again, similarly to OC43, BCoV preferentially binds a2,6-link SAs, but to a much lesser extent [218].

Canine respiratory coronavirus (CRCoV) was first detected in 2003 in dogs housed at a UK rehoming centre [281]. It is a betacoronavirus and a close relative of both OC43 and BCoV. Characterised by a dry, hacking cough, the disease is generally mild and self-limiting. However, it can progress to a potentially fatal bronchopneumonia [132,282]. CRCoV possesses an HE gene. Kienzle et al. [283] have identified the putative active site for esterase activity, FGDS, at amino acids 37–40 [283]. Due to its similarity with other coronavirus HE proteins, it is suggested to contribute to CRCoV receptor-binding and to act as a receptor destroying enzyme [284]. CRCoV was shown to be able to agglutinate chicken red blood cells indicating interaction of a viral surface protein with sialic acid residues on the erythrocyte surface by Schultze et al. [285]. Szczepanski et al. [218] also performed haemagglutination assays to verify whether CRCoV can use sialic acids as receptor molecules. To determine the importance of SAs for CRCoV attachment, cells were treated with neuraminidase (NA) prior to infection and then examined viral attachment. Removing SAs reduced attachment of CRCoV but did not affect viral replication, the same as demonstrated with both OC43 and BCoV. Contrastingly, CRCoV shows a preference for α2,3-SA [218]. This shows that sialic acid facilitates viral attachment but not entry. Due to the similarity of the CRCoV S glycoprotein to those of BCoV and HCoV-OC43, CRCoV is likely to bind to the same speculative receptors on the cell surface, namely 9-O acetylated sialic acid.

As with other betacoronaviruses of Lineage A viruses, PHEV readily interacts with a variety of red blood cells using it’s HE protein [133,286]. Specifically, PHEV attaches to N-acetyl-9-O-acetylneuraminic acid-containing receptors on erythrocytes [133]. Schultze et al. [133] produced purified HE protein from PHEV and identified that the HE protein retained its acetylesterase activity and was able to function as a receptor-destroying enzyme, rendering blood cells resistant to agglutination, but does recognise RBC surface receptors [133].

#### 9.3.2. The Effect of Sialic Acid Binding on the Tropism of Coronaviruses

With the extensive studying of Influenza virus interactions with SA, it has been demonstrated that the SA species that are bound by the virus is a major determinant of the host range and exhibited tropism [287]. SA binding and the resulting effect on the tropism of coronaviruses is most elegantly demonstrated by TGEV and PRCV. The S glycoprotein of TGEV is known to have two different binding activities, pAPN as discussed above and additionally, to sialic acids; allowing for agglutination of erythrocytes [288]. The two binding activities are located on different domains of the S glycoprotein with AA residues 145–209 (S1-NTD) important for the recognition of sialic acids and residues 522–744 (S1-CTD) for pAPN [156,157]. Sialic acid binding activity of TGEV is correlated with its enteropathogenicity [68,289]. PRCV unlike TGEV has no hemagglutinating activity [288] suggesting no sialic acid binding capability. The lack of SA binding activity is explained by a large deletion, ~600 nts, in the S gene that results in a truncated S glycoprotein, with a loss of almost the entire S1-NTD [68,290]. Research into TGEV identified several point mutations that result in the loss of SA binding and enteropathogenicity; these are located in the portion of S glycoprotein that is absent in PRCV. The loss of SA binding and subsequent loss of enteropathogenicity has resulted in altered tropism, with PRCV replicating with high efficiency in the respiratory tract but with very low efficiency in the gut [75]. However, PRCV retains the ability to bind pAPN.

IBV is the aetiological agent of infectious bronchitis (IB) [183,184,185,186,187]. Whereas the primary cellular receptors for the majority of coronaviruses is understood, the cellular receptor for IBV remains unknown but research has identified the possible receptor-binding domain for M41; a pathogenic IBV strain of the Massachusetts (Mass) serotype [252,291], to be located within the S1-NTD [254]. IBV is reported to use sialic acid (SA) as an attachment factor [100,253,264,292] and previous research utilising the S1 subunit of several strains of IBV suggests that the receptor interaction of the IBV S glycoprotein corresponds with pathogenicity and in vivo tropism [253]. It has been demonstrated that α2,3-linked sialic acid serves as a receptor determinant for IBV infection of Vero cells and primary chicken embryo kidney cells [264,293]. Results also show that α2,3-linked sialic acid also serves as a receptor determinant on chicken tracheal organ cultures. To date (24 January 2022), there is no data surrounding IBV binding to other conformations of sialic acid and is likely to be cell-type dependent.

## 10. Entry Receptors, Attachment Factors and Co-Factors: Other Binding Factors

### Transmembrane Protease Serine Type 2 (TMPRSS2)

The human protein transmembrane protease serine type 2 (TMPRSS2) (Figure 10C1) plays a crucial role in coronavirus infection, through activation of the S glycoprotein, facilitating entry into target cells, including MERS-CoV, SARS-CoV and SARS-CoV-2 [294,295]. It is required to prime the S glycoprotein through cleavage, allowing for endosome-independent entry into cells [29,105,294]. TMPRSS2 is a member of the type 2 transmembrane serine protease (TTSP) family, and is depicted by androgen receptor elements located beyond its transcription site [296]. As well as the cleavage and activation of the S glycoprotein TMPRSS2 is subjected to autocleavage, which results in the release of its soluble catalytic domain [297]. The conditions required for autocleavage of TMPRSS2 remains elusive [294]. It is expressed in lung and bronchial cells [298], in addition to the colon, stomach, pancreas, salivary glands and numerous other tissues [299]. It is co-expressed in bronchial and lung cells with the ACE-2 [298].

In recent studies, the proteolytic activation of the HCoV-229E S glycoprotein is analysed using trypsin-like serine proteases [300]. It is found that fusion activation is not dependent on the cleavage of the S1/S2 site, but is highly dependent on the cleavage in the S2′ region. This is very similar to the fusion activation of the IBV S glycoprotein, which requires furin-dependent cleavage at the S2′ site. A similar cleavage pattern was noticed upon co-expression of 229E-S with TMPRSS2 and HAT, indicating that these proteases and trypsin cleave the S-protein at similar or identical sites. The results suggest that TMPRSS2 and HAT cleave the 229E-S, likely at the same sites that are recognised by trypsin [301].

Shirato et al. [302] have shown that in Vero cells expressing TMPRSS2 (Vero-TMPRSS2) the susceptibility to MERS-CoV infection was 100-fold higher than that of non-TMPRSS2-expressing parental Vero cells. The serine protease inhibitor Camostat, which inhibits TMPRSS2 activity, also completely blocked syncytium formation but only partially blocked virus entry into Vero-TMPRSS2 cells [302]. MERS-CoV is thought to enter cells via two distinct pathways, one mediated by TMPRSS2 at the cell surface and the other mediated by cathepsin L in the endosome. Simultaneous treatment with inhibitors of both cathepsin L and TMPRSS2 completely blocked virus entry into Vero-TMPRSS2 cells, indicating that MERS-CoV employs both the cell surface and the endosomal pathway to infect Vero-TMPRSS2 cells [302].

As previously discussed, for membrane fusion, the SARS-CoV S glycoprotein relies on proteolytic activation at the S1/S2 boundary, where the S1 dissociates and S2 undergoes a major structural change [46,303]. These SARS-CoV entry-activating proteases include cell surface protease TMPRSS2 as well as lysosomal proteases–cathepsins [46,303]. Cleavage at the S2′ site is either by serine proteases (e.g., TMPRSS2) at the cell surface or by cathepsin proteases in the late endosome or endolysosome [304,305].

Protease activators for entry have been investigated for SARS-CoV-2 by Mahoney et al. [295]. Research demonstrates that that TMPRSS2 and lysosomal proteases are essential for SARS-CoV-2 entry [105,108,294]. TMPRSS2 is also an attractive therapeutic target for COVID-19 drug discovery [295]. Whether serine or cathepsin proteases are used for S2′ cleavage is cell-type dependent [105,295,305]. While entry into HAE and Calu-3 cells is cathepsin independent, entry into Vero cells, which do not express the required serine proteases, relies on cathepsins exclusively [105,295,305].

Contrastingly, towards the end of 2021 a SARS-CoV-2 highly transmissible variant of concern (VOC) emerged–referred to as the Omicron variant [306,307]. Peacock et al. [306] have demonstrated that the Omicron S glycoprotein has a reduced ability to induce syncytia formation when compared to other VOCs including the Delta variant. Additionally, they have highlighted that Omicron is able to efficiently enter cells via the endosomal route in a TMPRSS2-independent manner [306]. They suggest that it is this TMPRSS2-independent entry mechanism that allows Omicron to infect a larger number of cells in the respiratory epithelium, allowing higher infectivity at lower exposure doses, and therefore resulting in enhanced transmissibility [306]. Willet et al. [307] also identified the TMPRSS2-independent entry mechanism with both live virus cultures and viral pseudotypes favouring endosomal fusion. The data indicates that entry through endosomal fusion rather than the more traditional cell-surface mechanism could impact not only transmission, but cellular tropism and pathogenesis [307].

## 11. Unknowns of Coronavirus Receptor-Binding

Swine acute diarrhoea syndrome coronavirus (SADS-CoV), is a newly discovered, enveloped, positive-sense, single-stranded RNA virus belonging to the Alphacoronavirus genus [16,17,91,308]. SADS-CoV is considered to be the causative agent of the fatal swine acute diarrhoea syndrome (SADS) with clinical symptoms of severe, acute diarrhoea and rapid weight loss in piglets [16,17,91,308]. The SADS-CoV genome shares ~95% identity to that of bat alphacoronavirus HKU2, suggesting that it may have emerged from a natural reservoir in bats and crossed the species barrier [91]. As it is a newly emerged coronavirus, research has primarily focused on clinical diagnosis, molecular epidemiology, evolution and animal models [17,309,310,311]. The S glycoprotein of SADS-CoV (1130 amino acid residues in length) and is among the shortest coronavirus S glycoprotein lengths [312] and has an amino acid homology of less than 28% to other known coronavirus S glycoproteins (excluding HKU2), indicating that the spike gene of SADS-CoV is unique [16,17,308,309,311,313,314]. Whilst very limited, the receptor analysis indicates that none of the known coronavirus glycoprotein receptors including ACE-2, DPP4, or APN are essential for cell entry [17,91,315]. There are also no reports regarding the recognition of glycans by the NTD of SADS-CoV. It has been demonstrated that SADS-CoV is able to infect cells from a broad range of species including mouse, chicken, pig, monkey, and human, indicating a high potential for interspecies transmission [315].

Though the attachment factors and alternative receptors for IBV have been well documented, the primary receptor remains elusive. The varying tissue tropism and pathogenicity of IBV strains could mean that there is no universal primary receptor for IBV infection, and similarly to FCoV, the primary receptor could differ by serotype.

Additionally, although it is inferred that HKU-1 binding is mediated by sialic acids [269], it remains the only human coronavirus with an unidentified cellular receptor [269]. However, it has a documented attachment factor through the use of O-Ac-Sia to initiate the infection of host cells [269].

## 12. Conclusions and Perspectives

With the increasing interest in coronaviruses following on from the COVID-19 pandemic and the arrival of numerous variant viruses, it is becoming more and more vital that the cell entry mechanisms of coronaviruses are comprehensively understood. Several coronaviruses currently have both global and/or regional endemicity meaning that there is a very real threat of increased spread of novel coronavirus infections to both avian and mammalian species including humans. The ability of coronaviruses to be able to cross the species barrier with as little as two mutations (SARS-CoV from palm civets to humans), highlights the potential risk of other animal coronaviruses developing zoonotic tendencies. There is also the risk of spillover events, as demonstrated by BCoV in a bovine-to-human spillover event creating the seasonal human coronavirus OC43.

Documented research has identified bats as a reservoir for coronaviruses with zoonotic potential [12,316]. This was demonstrated through the emergence of SARS-CoV in 2003, with cross species transmission from bats, to palm civets, to humans [317]. It is also inferred that cross-species transmission from bats may have played a role in the emergence of SARS-CoV-2 in 2019. In the last decade, a number of additional SARS-like bat-borne coronaviruses have been identified, including RatG13, novel Bat CoVs identified in Laos (BANAL-236 and others) and Rs3367, with the latter sharing largely conserved regions surrounding the RBD [318]. Whilst RatG13 does not share the same degree of conservation, it remains the closest reported bat coronavirus to SARS-CoV-2 S glycoprotein [318]. A novel RatG13 strain identified in Laos in 2021 was identified as being the closest in origin to SARS-CoV-2 to date [319]. BANAL-236 S glycoprotein has a high affinity for hACE2 and pseudoviruses expressing the BANAL-236 S glycoprotein were able to efficiently enter human cells using an hACE2 dependent pathway [319]. Entry was also blocked using SARS-CoV-2 neutralising serum [319].

Receptor recognition is an important determinant of coronavirus infection and pathogenesis. It is also one of the most important targets for host immune surveillance and human intervention strategies. Receptor-binding preference is also key in the cell and tissue tropism exhibited by different viruses. The cell entry mechanisms of coronaviruses have implications for understanding clinical features of viral infections and whether viruses can evade immune surveillance and/or vaccination and therapeutic interventions. Altered receptor-binding preferences can lead to insufficient immune responses and extended recovery time. Better understanding of binding mechanisms of coronaviruses can benefit intervention strategies and identify novel and sometimes broad-spectrum antiviral targets.

Frequent arising variants and novel virus discoveries, coupled with the minimal host adaptation requirements for human infection, clearly identifies coronaviruses as posing a constant pandemic risk; whereby receptor-binding interactions should be scrutinised in order to combat coronavirus infection as efficiently as possible. Whilst the focus currently predominately fixates on human coronavirus infections and their subsequent proteinaceous receptor interactions, there are numerous circulating veterinary coronaviruses utilising lesser-studied protein receptors and glycans that continue to pose a threat to both human and animal health. This review has highlighted the number of sugar derived receptors that are widely used by several viruses across different genera; for example, neuraminic acid being utilised by TGEV, IBV, and MERS-CoV–and is an area that needs to be continuously studied in the same detail as receptors such as APN and ACE2.

## Data Availability

Not applicable.
